# Unmasking a Silent Threat: Improving Pulmonary Hypertension Screening Methods for Interstitial Lung Disease Patients

**DOI:** 10.3390/medicina60010058

**Published:** 2023-12-28

**Authors:** Vaida Averjanovaitė, Lina Gumbienė, Ingrida Zeleckienė, Virginija Šileikienė

**Affiliations:** 1 Faculty of Medicine, Vilnius University, LT-03101 Vilnius, Lithuania; ingrida.zeleckiene@santa.lt; 2 Clinic of Cardiac and Vascular Diseases, Faculty of Medicine, Institute of Clinical Medicine, Vilnius University, LT-03101 Vilnius, Lithuania; lina.gumbiene@santa.lt; 3 Clinic of Chest Diseases, Immunology and Allergology, Faculty of Medicine, Institute of Clinical Medicine, Vilnius University, LT-03101 Vilnius, Lithuania; virginija.sileikiene@santa.lt

**Keywords:** pulmonary hypertension, interstitial lung disease, cardiopulmonary exercise testing, lung function tests, echocardiography, cardiac magnetic resonance imaging

## Abstract

This article provides a comprehensive overview of the latest literature on the diagnostics and treatment of pulmonary hypertension (PH) associated with interstitial lung disease (ILD). Heightened suspicion for PH arises when the advancement of dyspnoea in ILD patients diverges from the expected pattern of decline in pulmonary function parameters. The complexity of PH associated with ILD (PH-ILD) diagnostics is emphasized by the limitations of transthoracic echocardiography in the ILD population, necessitating the exploration of alternative diagnostic approaches. Cardiac magnetic resonance imaging (MRI) emerges as a promising tool, offering insights into hemodynamic parameters and providing valuable prognostic information. The potential of biomarkers, alongside pulmonary function and cardiopulmonary exercise tests, is explored for enhanced diagnostic and prognostic precision. While specific treatments for PH-ILD remain limited, recent studies on inhaled treprostinil provide new hope for improved patient outcomes.

## 1. Introduction

Interstitial lung diseases (ILD) represent a heterogeneous group characterized by shared clinical, radiographic, and physiologic features. Pulmonary hypertension (PH) stands as a potential complication within this spectrum of ILDs. PH arising from ILD is classified as group 3 PH in accordance with the World Health Organization (WHO) classification. The diagnosis of group 3 PH now entails a resting mean pulmonary arterial pressure (PAP) > 20 mmHg, coupled with a pulmonary vascular resistance (PVR) ≥ 2 Wood units and a pulmonary artery wedge pressure (PAWP) ≤ 15 mm Hg at right-sided heart catheterization (RHC) in the context of chronic lung disease [1].

The progression of PH-ILD typically unfolds gradually, contributing to diminished exercise tolerance, respiratory insufficiency, and increased mortality rates [2]. To date, idiopathic pulmonary fibrosis (IPF) has been the most comprehensively investigated ILD in the context of PH. However, PH may manifest any ILD.

The precise prevalence of PH-ILD remains elusive, and estimates vary widely. Existing reports suggest that mild-moderate pulmonary arterial pressure elevation is quite common in ILD patients. Data from the COMPERA registry suggest that the presence of a PVR greater than ~5 Wood Units is associated with worse survival compared to a PVR ≤ 5 Wood Units in ILD patients [3]. Therefore, PVR can be used to distinguish between non-severe PH (PVR ≤ 5 WU) and severe PH (PVR > 5 WU). Evidently, non-severe PH frequently emerges in advanced ILD cases, whereas severe PH is a much rarer occurrence, affecting less than 10% of patients with advanced ILD [4,5]. It must be acknowledged that even non-severe PH in the context of lung disease is associated with worse survival, increased oxygen requirements, and a deterioration in functional status. Early detection and appropriate management can potentially improve patient outcomes and quality of life. 

This article aims to provide a comprehensive analysis of current diagnostic screening methods for PH-ILD. By discussing the mechanisms and clinical presentation of PH-ILD and exploring advanced diagnostic techniques of PH-ILD, our intent is to enhance understanding that informs clinical decision-making, thereby optimizing the management of PH-ILD.

## 2. PH-ILD Pathophysiology

The emergence of PH in the setting of ILD is caused by complex interactions within the lung parenchyma, vascular structures, and inflammatory pathways. In ILDs, destruction of the lung parenchyma triggers the localized release of an array of inflammatory and pro-proliferative cytokines, growth factors, and vasoactive agents [6,7,8,9]. These bioactive substances, in conjunction with increased vascular bed obliteration and vessel distortion due to fibrosis and lung parenchyma stiffening, collectively contribute to endothelial injury and smooth muscle hypertrophy. This intricate cascade ultimately leads to the development of PH [10]. Some experimental studies with rats suggest that changes to cardiovascular physiology start early in the development of lung fibrosis [11]. 

Histopathologic findings in explanted human lung tissue from PH patients with advanced fibrotic ILD indicate vascular wall thickening, luminal narrowing of the small pulmonary arteries and arterioles, plexiform lesions, medial hypertrophy, and fibrous vascular occlusions. Divergent findings exist concerning the extent of correlation between pulmonary vasculopathy and mean PAP [12,13].

Additionally, chronic hypoxia due to compromised gas exchange triggers further pulmonary vascular changes, including pulmonary vasoconstriction, vascular remodeling, and increased resistance within the pulmonary circulation [14]. This perpetuates a cycle of reduced tissue oxygenation and escalating hypoxemia, potentially contributing to the development of PH.

The presence of comorbidities such as left heart disease, sleep apnoea, pulmonary artery thromboembolism (PATE), and chronic obstructive pulmonary disease (COPD) can further contribute to PH development in ILD patients. These comorbidities can not only inflict additional damage on the pulmonary vasculature but also exacerbate the demands on an already compromised pulmonary blood flow.

A more detailed examination of PH-ILD pathophysiology is beyond the scope of this article, but it is apparent that numerous factors, ranging from local inflammatory mediators to systemic hypoxia and comorbidities, collectively contribute to the emergence of PH-ILD.

## 3. PH-ILD Disease Spectrum

PH can complicate any ILD, but the majority of data have been collected on PH related to idiopathic interstitial pneumonias (IIPs), each with distinctive radiographic and pathologic features. IPF, the most frequent subtype, belongs to this group, which also includes nonspecific interstitial pneumonitis (NSIP), desquamative interstitial pneumonitis, lymphocytic interstitial pneumonia, and cryptogenic organizing pneumonia. Other ILDs encompass exposure-related diseases like pneumoconiosis, asbestosis, silicosis, and drug-induced lung diseases from amiodarone, methotrexate, or chemotherapy use. A vital group comprises autoimmune/connective tissue disease-related diffuse lung diseases: rheumatoid arthritis, systemic lupus erythematosus, systemic sclerosis (SSc), polymyositis, dermatomyositis, Sjogren’s syndrome, or interstitial pneumonia with autoimmune features.

Granulomatous lung diseases, including sarcoidosis and chronic hypersensitivity pneumonitis, are significant, though PH associated with sarcoidosis falls under WHO group 5 PH due to multifactorial pathophysiology. About up to 30% of sarcoidosis patients are likely to experience PH [15]. Sarcoidosis characteristic granulomatous inflammation may affect the pulmonary arteries directly, but systemic disease effects likely also contribute to PH development [16]. Diastolic dysfunction is more common in sarcoidosis than in other parenchymal lung diseases, which can further raise PAP. Sarcoidosis can cause mediastinal lymphadenopathy that may mechanically impinge on the pulmonary arteries or involve the pulmonary veins. Additionally, sarcoidosis may affect the liver, contributing to porto-pulmonary hypertension. Current treatment for sarcoidosis-related PH primarily focuses on treating the underlying disease.

Unusual ILDs include pulmonary Langerhans cell histiocytosis (PLCH) and lymphangioleiomyomatosis (LAM). While PH prevalence is high in PLCH, it’s classified as group 5 PH; however, LAM-related PH is now in WHO group 3 [17]. This change was made after observations that PH in LAM tends to be mild, and the presence of PH is linked to compromised pulmonary function, indicating that the increase in PAP is associated with parenchymal involvement. 

Perhaps the most data on PH-ILD originate from IPF literature. The reported prevalence of PH in IPF varies, but it tends to increase with the progression of the disease [18]. PH in IPF is associated with poorer outcomes and shorter survival [19]. It often indicates a more advanced stage of the disease and a worse prognosis. Echocardiography cannot provide definitive hemodynamic measurements for diagnosing PH in IPF; rather, it offers an indication of the likelihood of PH. Nevertheless, even assessing this likelihood can be challenging in some IPF patients due to their poor ultrasonographic window. The gold standard diagnostic method, RHC, is typically reserved for lung transplantation candidates, making the diagnosis of PH in IPF a complex issue. Patients with IPF-related PH may experience increased dyspnoea, reduced exercise capacity, and a decreased 6-min walking test (6MWT) distance; some may require supplemental oxygen therapy [20]. The management of PH in IPF remains a topic of debate and mostly relies on underlying disease treatment and supportive measures.

Connective tissue diseases (CTDs) can fall into any of the 5 WHO groups based on primary phenotypes. Among CTDs, SSc is probably the most commonly associated with both pulmonary arterial hypertension (PAH) and ILD, with a prevalence ranging from 2 to 12% [21,22,23,24,25]. It is often difficult to distinguish SSc-related PH between group 1—PAH and group 3—PH due to the presence of parenchymal lung disease. The DETECT study proposed a promising diagnostic algorithm for SSc-PAH, but only RHC confirms the diagnosis [26]. Patients with preserved lung volumes can be safely treated with PAH drugs, but there’s less robust evidence for PH-SSc with advanced ILD. Accurate evaluation requires lung imaging alongside pulmonary function test (PFT) criteria. SSc patients with combined pulmonary fibrosis and PH face a high mortality risk of 8% at 1 year [27].

## 4. PH-ILD Phenotypes

Recently, the Pulmonary Vascular Research Institute’s Innovative Drug Development Initiative has outlined the importance of distinguishing distinct PH-ILD phenotypes characterizing differences in disease presentation, clinical course, and, possibly, treatment response [28]. Authors suggest that combined pulmonary fibrosis and emphysema (CPFE), PH associated with connective tissue and autoimmune diseases, PH associated with LAM, and post-tuberculosis PH represent different PH-ILD phenotypes with significant differences in their presentation and clinical course. 

PH seems to be a common complication among CPFE patients [29]. Furthermore, CPFE patients exhibit a greater severity of PH when compared to those with IPF [30] or COPD [31] alone. It has been hypothesized that the vasculopathy in cases of CPFE differs from what is seen in patients with COPD alone, and in the CPFE-related PH development, not only pulmonary hypertension WHO group 3 but also idiopathic pulmonary arterial hypertension (IPAH) might play a role. Vasculopathy in COPD has mainly been observed in small arteries and arterioles, whereas in CPFE, vasculopathy is broad and heterogeneous, involving arteries/arterioles, veins/venules, and capillaries [32]. There is typically relative preservation of airflow and lung volumes, as indicated by PFT; however, arterial oxygen levels and diffusing capacity for carbon monoxide (DLCO) levels tend to be markedly diminished [33]. 

In the context of autoimmune conditions, the clinical presentation of PH is shaped by a trio of interconnected pathological processes within the lung tissue: autoimmunity, fibrosis, and vasculopathy. PH frequently emerges as a complication in SSc and mixed connective tissue disease, typically falling under the classification of PH group 1—PAH. However, there are instances where SSc exhibits substantial pulmonary interstitial involvement, prompting some to classify it as group 3—PH. PH can also arise in cases of interstitial pneumonia with autoimmune features, where a form of ILD is associated with some, but not all, criteria for an autoimmune disease. Notably, PH appears to be a significant predictor of mortality in this patient group [34]. Research has shown that patients with pulmonary fibrosis and PH linked to an autoimmune disease experience improved survival rates after receiving PAH-targeted therapy, compared to PH-ILD patients without autoimmune disease [35]. Conversely, there were no apparent physiological differences in the response to pulmonary vasodilator treatment when comparing SSc-ILD patients with PH to SSc-PAH patients [27]. 

LAM is a cystic lung disease found almost exclusively in genetic females. In the updated PH classification, LAM-associated PH now resides within group 3. Several potential pathophysiological mechanisms in LAM-related PH development include vascular remodeling, infiltration of LAM cells into pulmonary artery (PA) walls, airflow obstruction, and hypoxia. Notably, a limited series of cases has unveiled instances of vascular remodeling and LAM cell infiltration into PA walls, offering valuable insights into the complexities of this disease [36]. Among LAM patients, those who develop PH often exhibit significantly reduced forced expiratory volume in 1 s (FEV1) and DLCO in comparison to those without PH [37,38]. The association between LAM and PH, as reflected in altered PFTs, suggests that the increase in mean PAP is predominantly linked to parenchymal involvement. It’s noteworthy that PH typically emerges during the advanced stages of the lung disease.

Approximately 20% of tuberculosis survivors experience persistent chronic respiratory issues encompassing various pathologies, including lung parenchymal fibrosis [39]. The prevalence and underlying mechanisms of post-tuberculosis PH remain largely unclear, making it challenging to classify within group 3 or group 5, akin to other granulomatous conditions like sarcoidosis (currently, post-tuberculosis PH is not listed in the WHO classification). Additionally, a connection between chronic thromboembolic pulmonary hypertension and prior tuberculosis has emerged, suggesting potentially even more intricate pathogenesis [40]. Notably, a history of smoking appears to define two subtypes of post-tuberculosis PH: COPD-like and ILD-like. The latter presents with poorer outcomes for patients, as evaluated using mean PAP and overall health status [39]. Importantly, a substantial proportion of post-tuberculosis PH cases arise in low- and middle-income countries, where access to comprehensive PH diagnostics may be limited, contributing to substantial patient care and knowledge gaps [41].

## 5. Challenges in Clinical Diagnosis of PH-ILD

Dyspnoea on exertion and fatigue often present as the predominant symptoms in both ILDs and early stages of PH. This overlap makes it impossible to rely solely on patients’ symptoms for predicting PH-ILD. Scientific literature documenting PH-ILD symptoms and quality of life is limited. Shortness of breath, fatigue, and swelling have been recognized as prevailing clinical manifestations among patients with PH associated with underlying lung disorders [42]. Additionally, cough emerges as a notable symptom, potentially exhibiting greater prevalence in PH linked to lung diseases compared to other PH cohorts. Furthermore, a substantial number of these patients articulate pronounced implications of the disease on their physiological, interpersonal, and psychological welfare. When signs of right heart failure emerge, PH suspicion is much more obvious, but that typically happens only in advanced ILD stages. Therefore, the need for PH screening is evident, especially in patients with disproportional symptom severity compared to parenchymal lung disease.

Thus, the suspicion of PH should be heightened when the progression of dyspnoea does not correspond with a decline in pulmonary function [43]. Profound hypoxemia and hyperventilation may also serve as indicators of potential PH development. However, it is crucial to consider other potential causes for worsening symptoms in ILD patients, such as venous thromboembolism, exacerbation of underlying ILD, left heart disease, or infection.

## 6. PH-ILD Diagnostic Strategy

The gold standard for diagnosing PH is RHC. When it is conducted in experienced PH centers, the incidence of procedure-related serious adverse events and mortality was shown to be very low [44]. Nonetheless, due to the invasive nature of the procedure, it should be reserved for cases where the results could significantly impact disease management. This includes enrolling patients in lung transplant lists, clinical trials, or initiating treatment with pulmonary vasodilators.

Currently, there is no universally accepted non-invasive algorithm to guide the selection of ILD patients who require further PH investigation. Since many PH-ILD patients do not routinely undergo RHC, it is crucial to assess the effectiveness of various individual and combined non-invasive tests for selecting patients necessitating further PH-ILD evaluation.

Diagnosing PH-ILD solely based on the patient’s medical history, physical examination, and PFTs is inadequate. However, certain non-invasive tests commonly conducted in patients with severe pulmonary and cardiovascular conditions may offer insights into the likelihood of PH-ILD. Some of the imaging tests commonly used in ILD patients and possible PH-indicating features are presented in Table 1. Presently, PH guidelines recommend interpreting echocardiographic parameters in conjunction with arterial blood gas (ABG) analysis, PFTs, and computed tomography (CT) imaging when suspecting PH in lung disease patients [1]. In the subsequent sections, we will provide an overview of non-invasive testing methods for PH-ILD. 

### 6.1. Pulmonary Function Testing

PFTs are crucial for ILD management and assessing disease progression. Studies suggest that spirometric and plethysmographic parameters lack diagnostic value for PH-ILD [45]. However, certain trends in PFT results can provide valuable insights into the presence of PH. Evidently, decreased DLCO in the presence of relatively preserved lung volumes could potentially indicate the presence of PH [46]. A combination of DLCO < 40% and the need for oxygen therapy has been observed to be specific, though not very sensitive, for identifying PH in the IPF population. Furthermore, a clinical scoring system consisting of DLCO, PA/Ao ratio on chest CT, and PaO_2_ has been demonstrated to predict the elevation of mean PAP in patients with IPF [47].

The FVC/DLCO ratio has been proposed as a potential predictor of PH in the setting of ILD [48]. The premise is that a disproportionately lower DLCO as compared to FVC could indicate the development of PH. However, an established threshold value for FVC/DLCO is lacking, and the applicability of this indicator may be diminished in the presence of emphysema (combined pulmonary fibrosis and emphysema—CPFE) [49].

### 6.2. Cardiopulmonary Exercise Testing

Resting PFTs often cannot reliably predict exercise performance or exertion-related dyspnoea. Cardiopulmonary exercise testing (CPET) provides a precise objective and quantitative assessment of a patient’s cardiovascular and ventilatory responses during exercise, unveiling integrated anomalies in respiratory, cardiovascular, metabolic, muscular, and neurosensory systems in ILD [50]. Additionally, it might offer insights into some of the pathophysiological abnormalities imposed using PH-ILD. In individuals with group 3 PH, CPET often shows significantly impaired maximal aerobic capacity.

There is evidence that the ventilation/carbon dioxide (VE/VCO_2_) slope may assist in predicting PH in IPF patients, where the area under the receiver operating characteristic curve was 0.94 (0.89–0.98) [45]. In IPF patients with PH, significant correlations have been reported between systolic PAP and several CPET parameters, such as peak oxygen uptake (VO_2_), anaerobic threshold (AT), and peak O_2_ pulse [51]. In another study, the optimal cut-off value for predicting a resting systolic PAP ≥ 40 mmHg for IPF patients was a VE/VCO_2_ at an anaerobic threshold of > 45 [52]. PeakVO_2_ and VE/VCO_2_ slope have demonstrated prognostic value in predicting survival among SSc patients and may also enhance the pre-test probability of PH [53].

While CPET is valuable for evaluating ventilatory or cardiac limitations in patients with chronic lung diseases, its applicability for PH-ILD diagnostics requires further exploration.

### 6.3. 6 min Walking Test

As ILD patients’ exercise tolerance tends to deteriorate, the presence of pulmonary vasculopathy further exacerbates this impairment. One of the most widely used methods for assessing changes in exercise tolerance is the 6MWT. During this test, parameters including walking distance, oxygen saturation, heart rate, and Borg dyspnoea scale are recorded. 

Lower oxygen saturation observed during the 6MWT and a slower heart rate recovery within the first minute of rest subsequent to the test could serve as potential indicators of PH-ILD. A persistently elevated heart rate one minute subsequent to a 6MWT holds predictive value for PH, demonstrating a low sensitivity of 52% and a moderate specificity of 74% [54]. Final oxygen saturation by pulse oximetry (SpO_2_) < 88% on 6MWT has been reported to predict significantly worse survival in patients with PH-ILD [55]. Significantly, PH-ILD seems to contribute to exercise intolerance independently from the extent of fibrosis, and PVR is a strong predictor of 6MWT distance [56]. 

On the other hand, it is worth noting that a decrease in any of the 6MWT parameters could indicate not only the vascular component of PH but also the involvement of the parenchymal disease. Thus, while 6MWT offers valuable insights into exercise tolerance and potentially PH-ILD diagnosis, its results need to be interpreted with caution, taking into consideration the intricate interplay between vascular and lung parenchymal factors in these patients. 

### 6.4. Chest Computed Tomography

Chest CT is routinely performed in ILD patients. It also plays a crucial role in the initial assessment of PH [57]. When the cause of PH is uncertain, chest CT could be helpful in differentiating etiology [58]. 

Chest CT can provide early diagnostic insights into PH by characterizing cardiovascular and parenchymal changes [59]. Notably, dilation of the main pulmonary artery (PA) is a prevalent chest CT finding in PH cases. One of the foundational studies in this field reported that a PA diameter exceeding 29 mm has a sensitivity of 75% and specificity of 89% for PH presence [60]. Subsequent studies identified various PA diameter thresholds: ≥28 mm as sensitive, ≥30 mm as a compromise, and ≥32 mm as a specific PH indicator [61]. An elevated ratio of PA to ascending aorta diameters is another CT-based predictor of PH [62]. 

However, dilated PA should be interpreted with caution in ILD patients. Controversy arises from contrasting outcomes, with some authors suggesting that fibrosis-induced traction could lead to PA distention without PH [63]. Additionally, ILD exacerbations could also impact PA diameter [64]. 

**Figure 1:**
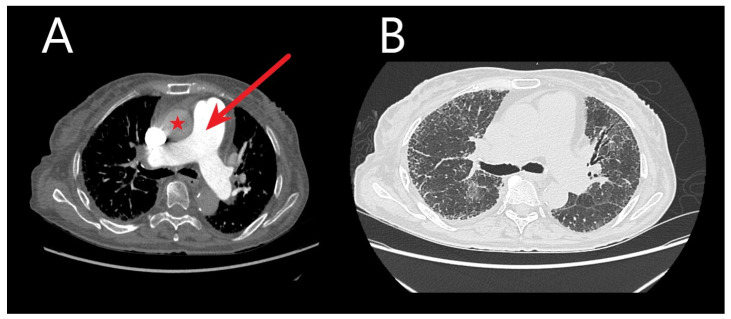
Chest computed tomography (CT) images without and with contrast are shown from an idiopathic pulmonary fibrosis patient. (**A**) Transaxial images are shown demonstrating an enlarged main pulmonary artery (red arrow) compared to the ascending aorta (red star) at the same level—suggestive of pulmonary hypertension. (**B**) Transaxial images in the lung window demonstrate bilateral honeycombing, intralobular, and interstitial thickening.

CT reveals cardiac changes reflective of chronic pressure overload in PH progression (Figure 1). These changes encompass right ventricular (RV) hypertrophy (wall thickness > 5 mm), dilated RV with an RV/left ventricle (LV) ratio > 1.0 in the axial plane, flattened interventricular septum, and dilated inferior vena cava [65]. Normally, the RV exhibits thin walls (4–5 mm), with less than one-third the thickness of the LV on chest CT. Increased RV:LV ratio and interventricular septal angle reflect RV enlargement and pressure overload in pre-capillary PH [61] but perhaps might be more accurately evaluated on gated cardiac MRI. 

An “egg and banana” sign is recognized as indicative of PH, characterizing the PA lateral to the aortic arch, with the aortic arch likened to a banana and the PA to an egg [66]. Certain features like vascular tortuosity, mural calcification, vascular remodeling, and “pruning” of peripheral branches have been described as classic long-standing PH indicators. Pericardial thickening or effusion and contrast reflux into the inferior vena cava are noted as potential CT signs of moderate-severe PH [67]. CT imaging also might provide prognostic information, but its role in follow-up is limited by exposure to radiation [68]. 

### 6.5. Echocardiography

**Figure 2:**
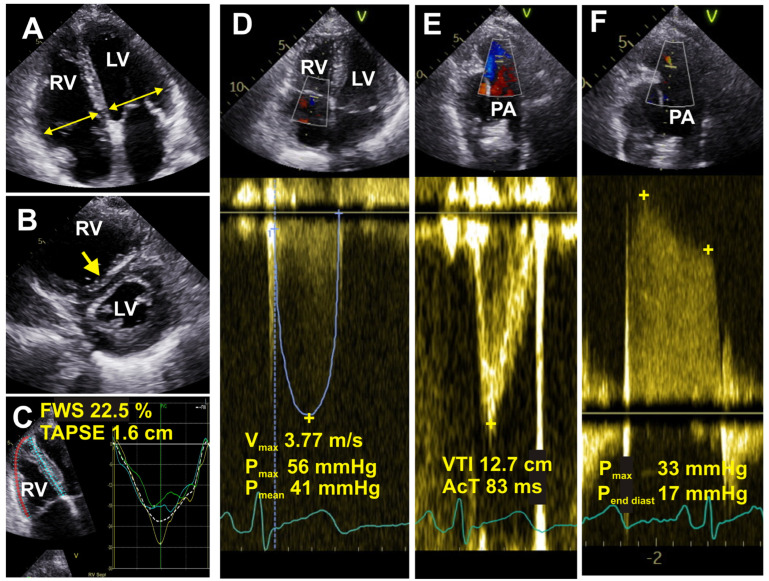
Echocardiographic images are shown in a patient with idiopathic pulmonary fibrosis. (**A**) Four chamber view: the maximum diameters of the right (RV) and left (LV) ventricles are measured (yellow arrows). Dilated RV with a ratio to left ventricle at about 1.0 is shown. (**B**) Parasternal short-axis view: large RV, flattened intraventricular septum (yellow arrow). (**C**) Right ventricular speckle strain, four chambers adjusted for RV: tricuspid annular plane systolic excursion (TAPSE) is reduced at 1.6 cm, and free wall right ventricular strain (FWS) is also reduced at 22.5%. (**D**) Four chamber view: septal deviation towards LV showing evidence of elevated RV pressure. Continuous wave Doppler through the tricuspid valve and Vmax is elevated at 3.77 m/s. (**E**) Pulse wave Doppler through right ventricular outflow tract (RVOT) indicating shortened pulmonary artery (PA) acceleration time (AcT) at 83 ms. (**F**) Continuous wave Doppler: PA regurgitation, estimated end diastolic PA pressure is elevated at 17 mmHg.

Echocardiography is probably the most widely used non-invasive diagnostic tool for assessing PH (Figure 2). It provides high spatial resolution and adequate morphological, functional, and hemodynamic analysis. However, the information obtained from echocardiography can only grade the probability of PH rather than delivering a conclusive diagnosis.

The echocardiographic estimation of PH primarily hinges on the peak tricuspid regurgitation velocity (TRV). A peak TRV > 2.8 m/s is indicative of PH, although comprehensive evaluation requires consideration of other parameters pertaining to RV morphology and function. If there is a good-quality signal and TRV is greater than 3.4 m/s, it shows a high probability of PH being present even without other echocardiographic markers [69]. Key echocardiographic indices used to evaluate the likelihood of PH in patients with advanced lung diseases include the tricuspid annular plane systolic excursion (TAPSE), RV systolic pressure, and RV outflow tract diameter [70].

When performing transthoracic echocardiography, ultrasound waves must first traverse several bodily layers, including skin, subcutaneous fat tissue, ribs, and lungs, before being reflected off the heart and captured by the probe to form an image. All of these structures, along with fibrotic lung parenchyma, can attenuate the ultrasound signal during its journey, potentially diminishing image quality. Thus, the “poor acoustic window” is often encountered in ILD patients [71], and it may pose challenges in accurately assessing the TRV.

It is worth mentioning that TRV can only be measured in the presence of detectable tricuspid regurgitation. While tricuspid regurgitation is usually present in severe PH, it may pose limits in mild or moderate PH. Some studies suggest that TRV might be unmeasurable in >50% of patients with chronic lung diseases, and there seems to be a propensity for overestimating PAP [72]. In instances where extensive lung parenchymal disease is present, the use of alternative echocardiographic measures suggests the probability of PH, such as decreased TAPSE, enlarged RA area, an increased RV:LV ratio, and LV eccentricity have been recommended. 

Additionally, newer echocardiographic methodologies such as three-dimensional (3D) assessment of the RV and RV longitudinal strain using 2D speckle tracking show promise in evaluating patients with PH-ILD [68]. Some authors have also explored the potential utility of transesophageal echocardiography to diagnose and measure RV systolic pressure [73]. Perhaps the most notable limitation of echocardiography is operator dependence, and although it has undeniable clinical value in diagnosing PH, it cannot yet replace RHC [74].

### 6.6. Cardiac Magnetic Resonance Imaging 

Cardiac MRI offers a comprehensive assessment of morphological and hemodynamic-functional parameters of the PA and RV, making it valuable for PH diagnosis and monitoring [68]. 

**Figure 3:**
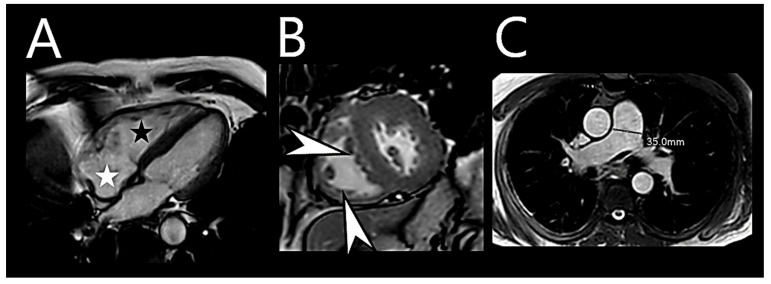
Cardiovascular magnetic resonance imaging in pulmonary hypertension. (**A**) End-diastolic 4-chamber view of a patient with pulmonary hypertension. There is dilation of the right ventricle (black star) and atrium (white star), with leftward septal bowing and hypertrophy of the right ventricular free wall and trabeculations. (**B**) Short-axis slice of the same patient. A stack of short-axis slices enables the quantification of volumes and mass. There is leftward septal bowing and hypertrophy of the right ventricular free wall and trabeculations (white arrow). (**C**) Pulmonary trunk in relation to the aorta. The pulmonary trunk and the right pulmonary artery are dilated.

Various MRI parameters, including interventricular septal bowing, LV eccentricity, ventricular mass index, asynchrony, RV ejection fraction, and RV end-systolic volume index, have been utilized to evaluate overall hemodynamic condition and aid in PH diagnostics (Figure 3) [75,76,77,78]. 

MRI evaluation extends beyond diagnosis; it also holds prognostic potential. Studies have indicated that RV ejection fraction and RV end-systolic volume index can predict PAH mortality [79], while indices reflecting RV structure and stiffness of the proximal pulmonary vasculature independently prognosticate PAH [80]. Furthermore, cardiac MRI indices, notably the percentage-predicted RV end-systolic volume index, show promise for PAH risk stratification, particularly when used in conjunction with other risk assessment approaches [81]. Furthermore, adjustment of RV functional measurements for age and sex may improve prognostication.

**Figure 4:**
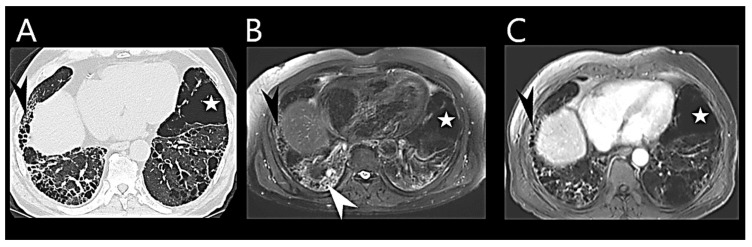
Cardiovascular magnetic resonance imaging (MRI) in idiopathic pulmonary fibrosis. Comparison of radiological findings in idiopathic pulmonary fibrosis with thin-slices multi-detector computed tomography (MDCT) and MRI. Thin-slices MDCT (**A**), MRI STIR (Shot Tau Inversion Recovery) sequence (**B**), and T1-weighted contrast-enhanced administration (at 10 min.) (**C**). The magnified images of MDCT and MRI show reticulation and honeycombing (black arrow) as well as areas of emphysema (white star). The high signal of ground-glass opacity in MRI (white arrow, (**B**)) may be due to water content and indicates active inflammation (white arrow, (**B**)).

Multivariate MRI models have been developed to accurately estimate PAP and differentiate disease severities in suspected PH patients [82,83]. Some models designed for identifying PH in chronic lung disease patients rely on parameters such as interventricular septal angle, ventricular mass index, and diastolic pulmonary artery area [84,85]. There are multiple novel and evolving MRI imaging approaches that report on the RV and might prove invaluable for earlier non-invasive PH diagnosis [86]. Recently, MRI has been emerging as an imaging modality to assess ILD (Figure 4).

Nonetheless, it’s important to note that factors such as cost, availability, long scanning times, and patient discomfort due to scanner noise or claustrophobia may present obstacles to the widespread utilization of MRI for early PH diagnosis. 

### 6.7. Biomarkers

No single ideal biomarker has been identified for the detection of PH, but some have shown promise in providing informative insights. To date, most research has concentrated on biomarkers for diagnosing group 1 and IPAH. Among the novel biomarkers that appear to be particularly informative and potentially useful are red cell distribution width (RDW), osteopontin, angiopoietin-2, endoglin, endostatin, and growth differentiation factor 15 (GDF-15) [87].

However, there’s considerably less evidence available concerning biomarker utilization in other PH groups, including group 3 and PH-ILD. A study involving 212 ILD patients suggests that N-terminal pro-brain natriuretic peptide (NT-proBNP) values below 95 ng/L can help rule out PH in ILD cases. Conversely, elevated uric acid levels were associated with the presence of PH, and uric acid, troponin T, and D-dimer levels were linked to a worse prognosis [88]. Some other biomarkers that have shown predictive value for PH in IPF include CXC-chemokine ligand 13 (CXCL13), a chemokine associated with B-cell homing. A 2014 publication reported higher plasma concentrations of this protein in IPF patients with PH and those with more severe disease [89]. Additionally, a molecule associated with immune and oxidative stress pathways, namely S100A12 (also known as Calgranulin C or EN-RAGE), was discovered to be elevated in peripheral blood mononuclear cells of individuals with pulmonary fibrosis-related PH [90].

While plasma levels of brain natriuretic peptide (BNP) and NT-proBNP are often elevated in all PH patients, including those with lung diseases, their sensitivity is limited as they frequently reflect left heart disease (LHD), a common comorbidity in ILD that can contribute to cor pulmonale. However, when combined with other parameters, BNP and NT-proBNP demonstrate value in PH-ILD screening [91]. For example, an algorithm that incorporates BNP, DLCO, and echocardiography has shown promising results as an indicator of PH-ILD [92]. Overall, BNP and NT-proBNP undoubtedly remain valuable tools for general PH screening and risk stratification.

## 7. Artificial Intelligence Applications

Artificial Intelligence (AI) refers to the simulation of human intelligence in machines or computer systems, enabling them to perform tasks that typically require human cognitive abilities. Within AI, Machine Learning (ML) represents a subset of AI that focuses on developing algorithms and models capable of enabling computers to learn from extensive datasets, commonly referred to as “big data”. The application of AI in medical diagnostics holds significant promise for improving the precision, efficiency, and speed of identifying a spectrum of medical conditions, including PH. 

Kogan and colleagues have made notable contributions to this field, as demonstrated by their recent work [93]. They have designed an ML model capable of detecting PH using electronic health records. The results of their study show the model’s capability to accurately retrospectively predict the diagnosis of PH up to 18 months prior to the clinical confirmation of PH. It proves the potential of ML tools to substantially reduce diagnostic delays in PH and thereby enhance patient outcomes.

Several other ML models with varying degrees of accuracy have also been developed for PH. Some focus on specific diagnostic tests [94,95], while others target specific subgroups within the PH population [96]. Generally, the development and utilization of ML models in PH and potentially PH-ILD diagnosis offer promising prospects for improving patient care and outcomes. 

## 8. Management and Treatment

Currently, approved medical treatment targeted specifically for PH-ILD is not available in most countries. Patients should receive optimal treatment for their underlying ILD, although a detailed summary of treatment recommendations for the diverse range of ILDs is beyond the scope of this article. Supportive care, long-term oxygen therapy (LTOT), treatment for sleep-disordered breathing, and alveolar hypoventilation may provide benefits [46]. To our knowledge, there are no studies specifically addressing the impact of LTOT on PH-ILD. However, most ILD international guidelines recommend long-term oxygen therapy for patients with stable severe daytime hypoxemia (arterial oxygen tension <56 mmHg) or less severe resting hypoxemia (PaO_2_ 56–59 mmHg) with evidence of hypoxic organ damage, including PH or right heart failure [97]. Additionally, enrolment in pulmonary rehabilitation programs can enhance patients’ quality of life [98]. In some cases, lung transplantation may be indicated [99], although it is typically reserved for a minority of ILD patients due to their advanced age and significant comorbidities.

Although anti-fibrotic drugs, effective for IPF and certain other progressive fibrotic ILDs, improve FVC and slow disease advancement, their impact on PH development requires additional investigation. Standard use of medications approved for treating PAH is not advised for the treatment of PH-ILD patients as their safety and effectiveness in this particular patient group remain uncertain [100]. Nevertheless, registry data suggest occasional prescriptions on an individual basis.

Most clinical trials investigating pulmonary vasodilators in PH-ILD have produced unsatisfactory outcomes [101]. However, some experts contend that these negative results might have been influenced, at least in part, by trial design and enrolment issues. The rarity of patients and their limited life expectancy further complicates trial completion [46]. The outcomes of studies examining the efficacy of sildenafil in treating PH-ILD have been largely disappointing [102]. Likewise, investigations into the potential PH-ILD treatment using ambrisentan or riociguat raised some concerns due to adverse safety signals. Specifically, ambrisentan was associated with worsened clinical outcomes in IPF patients, and the ARTEMIS-IPF trial was terminated early [103]. RISE-IIP study showed that riociguat carries an elevated risk for increased serious adverse events and mortality in IIPs-related PH [104]. As a result, neither of these medications is recommended for the treatment of PH-ILD [1].

Recent studies offer a glimpse of hope for PH-ILD-specific treatment. Treprostinil is a tricyclic benzidine analog of epoprostenol, which promotes direct arterial vasodilation and the inhibition of platelet aggregation. The INCREASE trial evaluated inhaled treprostinil versus placebo in PH-ILD patients [105]. Those treated with inhaled treprostinil demonstrated significant improvements in exercise capacity measured using the 6MWT. Study subjects receiving treprostinil demonstrated a reduced frequency of clinical deterioration in contrast to those in the placebo group. Representing a noteworthy development, the US Food and Drug Administration endorsed treprostinil for PH-ILD treatment in 2021. The 2022 ESC/ERS PH guidelines suggest considering inhaled treprostinil for PH-ILD patients, with further data required to evaluate long-term outcomes (recommendation class IIb, level B) [1].

**Figure 5:**
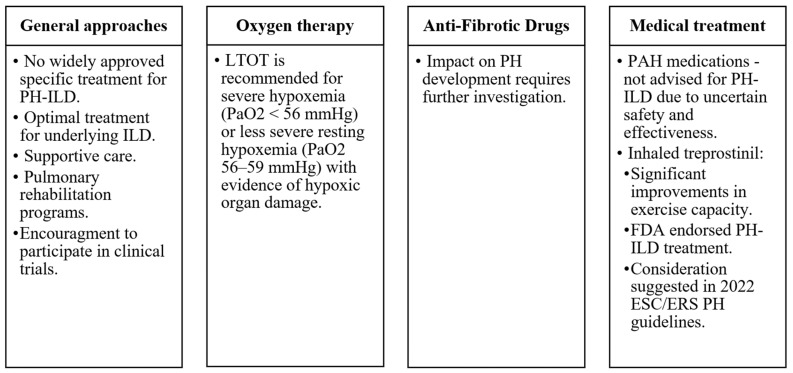
Pulmonary hypertension care essentials in interstitial lung disease patients [1]. Abbreviations: ILD—interstitial lung Disease, LTOT—long-term oxygen therapy, FDA—the US Food and Drug Administration, PAH—pulmonary arterial hypertension, PH—pulmonary hypertension, PH-ILD—pulmonary hypertension associated with interstitial lung disease.

Administering medication via inhalation holds promise as a treatment approach for PH-ILD patients and may lead to increased drug concentrations in adequately ventilated lung regions, consequently mitigating ventilation-perfusion mismatch. However, a phase 3 clinical trial assessing inhaled nitric oxide (iNO) as another potential PH-ILD treatment did not meet its primary endpoint and was terminated in July 2023, available at https://www.clinicaltrials.gov/study/NCT03267108?intr=NCT03267108&rank=1 (accessed on 27 October 2023). The guidance remains that PH-ILD patients should be considered for participation in clinical trials whenever possible (Figure 5).

## 9. Prognosis and Outcomes

The prognosis for many progressive fibrotic ILDs is inherently unfavorable. Nonetheless, the emergence of PH can inflict an even more damaging impact on disease course. It is evident that the development of PH in ILD patients is associated with worse functional status, decreased quality of life, and reduced survival rates. Notably, elevated mean PAP values, as measured using RHC during the initial assessment of IPF patients, have been reported as an independent prognostic indicator for survival [106]. Moreover, even mild PH has been demonstrated to significantly increase mortality risk among patients with IPF awaiting lung transplantation [107]. Higher mean PAP at the initial evaluation has a statistically significant impact on survival for patients with lung-dominant connective tissue disease [108]. It has been reported that there exists an annual increase of 1.8 mm Hg per year in mean PAP among IPF patients presenting with PH [2], thereby illustrating a progressively deteriorating characteristic. In contrast, the reported rate for COPD-related PH stands at 0.4 mm Hg per year [109], suggesting potential ramifications of ILD that are even more profound when juxtaposed with those of COPD.

## 10. Conclusions

PH has emerged as a relatively common and debilitating complication among patients with ILDs. Due to symptom overlap with ILDs and the compromised overall condition of these patients, obtaining precise diagnostic accuracy for PH-ILD can often be challenging. To progress toward a PH-ILD diagnosis, a comprehensive approach is essential, and a definitive confirmation demands RHC. Continued research and a deeper understanding of the interplay between PH and progressive ILDs are critical for improving our diagnostic and treatment strategies, enabling us to provide better patient care, improve disease outcomes, and ultimately achieve a better quality of life for PH-ILD patients.

## 11. Key Points

- PH-ILD encompasses a range of conditions, each with unique differences in clinical presentations and characteristics.
- Increased suspicion of PH is warranted when the progression of dyspnoea does not align with a decline in pulmonary function parameters.
- Diagnosing PH-ILD is challenging due to symptom overlap with ILDs without PH, emphasizing the need for non-invasive tests like echocardiography, cardiac MRI, and specific biomarkers. For now, RHC remains the gold standard for confirming PH-ILD diagnosis.
- RHC is typically conducted primarily for ILD patients who meet the criteria for lung transplantation, are involved in clinical trials, or are being evaluated for specialized treatment.
- There are currently limited specific treatments for PH-ILD, but recent trials with inhaled treprostinil show promise.
- Managing PH-ILD involves supportive care, long-term oxygen therapy, and, in some cases, lung transplantation, with a focus on improving patient quality of life and prognosis.

## Figures and Tables

**Table 1:** Imaging features suggestive of pulmonary hypertension in patients with interstitial lung disease.

Imaging Modality	PH Suggesting Features
Chest CT	- Enlarged PA diameter - RV hypertrophy and dilation - Dilated inferior vena cava - “Pruning” of peripheral branches - Contrast reflux into inferior vena cava
Echocardiography	- TRV > 2.8 m/s - Decreased TAPSE - Enlarged RA area - Increased RV:LV ratio - Flattened interventricular septum
Cardiac MRI	- Enlarged PA diameter - RV hypertrophy and dilation - Reduced RV ejection fraction - Increased LV eccentricity index - Increased ventricular mass index

Abbreviations: CT—computed tomography, LV—left ventricle, MRI—magnetic resonance imaging, PA—pulmonary artery, PH—pulmonary hypertension, RV—right ventricle, TRV—tricuspid regurgitation velocity.

## Data Availability

No new data were created.

## Abbreviations

6MWT	6-Minute Walk Test
ABG	Arterial Blood Gas
AI	Artificial Intelligence
AT	Anaerobic Threshold
BNP	Brain Natriuretic Peptide
CPET	Cardiopulmonary Exercise Testing
CT	Computed Tomography
CXCL13	CXC-Chemokine Ligand 13
COPD	Chronic Obstructive Pulmonary Disease
CPFE	Combined Pulmonary Fibrosis and Emphysema
CTDs	Connective Tissue Diseases
DLCO	Diffusing Capacity for Carbon Monoxide
FEV1	Forced Expiratory Volume in 1 Second
FVC	Forced Vital Capacity
GDF-15	Growth Differentiation Factor 15
ILDs	Interstitial Lung Diseases
IIPs	Idiopathic Interstitial Pneumonias
iNO	Inhaled Nitric Oxide
IPAH	Idiopathic Pulmonary Arterial Hypertension
IPF	Idiopathic Pulmonary Fibrosis
LAM	Lymphangioleiomyomatosis
LHD	Left Heart Disease
LTOT	Long-Term Oxygen Therapy
LV	Left Ventricle
ML	Machine Learning
NSIP	Nonspecific Interstitial Pneumonitis
PA	Pulmonary Artery
PAH	Pulmonary Arterial Hypertension
PAWP	Pulmonary Artery Wedge Pressure
PAP	Pulmonary Arterial Pressure
PATE	Pulmonary Artery Thromboembolism
PH	Pulmonary Hypertension
PH-ILD	Pulmonary hypertension associated with Interstitial Lung Disease
PLCH	Pulmonary Langerhans Cell Histiocytosis
PVR	Pulmonary Vascular Resistance
RDW	Red Cell Distribution Width
RHC	Right-sided Heart Catheterization
RV	Right Ventricle
SSc	Systemic Sclerosis
TAPSE	Tricuspid Annular Plane Systolic Excursion
TRV	Tricuspid Regurgitation Velocity
VE/VCO_2_	Ventilation/Carbon Dioxide
VO_2_	Oxygen Uptake
WHO	World Health Organization
